# Spatial and Temporal Distribution of Particulate Phosphorus and Their Correlation with Environmental Factors in a Shallow Eutrophic Chinese Lake (Lake Taihu)

**DOI:** 10.3390/ijerph15112355

**Published:** 2018-10-25

**Authors:** Ming Kong, Jianying Chao, Wei Zhuang, Peifang Wang, Chao Wang, Jun Hou, Zhaoshi Wu, Longmian Wang, Guang Gao, Yu Wang

**Affiliations:** 1 Nanjing Institute of Environmental Sciences, Ministry of Environmental Protection, No. 8 Jiang Wang Miao Street, Nanjing 210042, China; kongming@nies.org (M.K.); zhuangwei@nies.org (W.Z.); wlm@nies.org (L.W.); 2 Key Laboratory of Integrated Regulation and Resources Development on Shallow Lakes, Ministry of Education, Hohai University, Nanjing 210098, China; njauchao@163.com (C.W.); hjy_hj@hhu.edu.cn (J.H.); 3 State Key Laboratory of Lake Science and Environment, Nanjing Institute of Geography and Limnology, Chinese Academy of Sciences, Nanjing 210008, China; zswu1987@163.com (Z.W.); gguang@163.com (G.G.); 4 Machinery and Equipment Industry Park Management Committee of Harbour Economic Development District, Jiangyin City 214400, China; wangyuwangyu@163.com

**Keywords:** particulate phosphorus, suspended particulate matter, species, ^31^P NMR, eutrophication

## Abstract

Spatial and seasonal variations of particulate phosphorus (PP) in a large shallow, eutrophic Lake Taihu with different ecotypes (including a phytoplankton-dominated zone, lake center zone, estuary zone and macrophyte-dominated zone) were investigated. The results showed that particulate organic phosphorus (POP) was the dominant form of PP (>88.0%). The concentration of POP showed higher levels in the bloom-sensitive northwestern zone (phytoplankton-dominated zone and estuary zone) during warm seasons, phytoplankton blooms and input of exogenous particulate matter were the main sources of POP in the lake water. Based on ^31^P nuclear magnetic resonance (^31^P NMR) analysis, orthophosphate (Ortho-P) was the dominant molecular species of PP and positively correlated with soluble reactive phosphorus (SRP) (*p* < 0.01). This suggested that the release of Ortho-P from suspended particulate matter (SPM) was the main source of SRP in the lake water. Pyrophosphate (Pyro-P), which is regarded as a highly labile species of P compounds, represented a large fraction of PP, and its significant positive correlations with chlorophyll *a* (Chl *a*), indicated that the concentration of Pyro-P could be used as an important indicator for the degree of eutrophication of Lake Taihu. These results proved that PP in lake water was a significant factor supporting lake eutrophication and must be controlled.

## 1. Introduction

Phosphorus (P) is an important driver of primary production in surface waters. Excessive P concentration is considered the most common cause of eutrophication [1], so it needs to be managed to avoid or reduce eutrophication. In aquatic systems, P species are found in dissolved and particulate fractions [2]. Particulate P (PP) often accounts for a larger proportion of total phosphorus than dissolved P [3], and acts as a potential source of dissolved P in lacustrine environments. Furthermore, PP is divided into particulate inorganic P (PIP) and particulate organic P (POP) [4]. PIP occurs in mineral phases, such as orthophosphate (Ortho-P), pyrophosphate (Pryo-P) and polyphosphate (Poly-P), which can be adsorbed to biotic and abiotic particles and as intracellular storage products [5]. POP comprises P incorporated in living and detrital organic molecules such as phosphomonoesters (Mono-P), phosphodiesters (Diester-P) and phosphonates [6]. PP in different species shows distinct exchangeability, reactivity and bioavailability, which can play different roles in the biogeochemical cycling of P in aquatic environments. Hence, it is necessary to research the characteristics of each species of PP, because this is useful for understanding their cycles in aquatic environments.

In lake water, suspended particulate matter (SPM) is the most important carrier of PP, which is mainly derived from the input of exogenous particulate matter and suspended sediment [7]. SPM in lakes mainly includes algae residues, clay minerals and plant debris, etc. [8], and its composition is related to the ecological types of lake zones. This determines that the levels and species of PP is varied in lake zones with different ecotypes. The cycling of PP is a dynamic process in lake ecosystems. The species of PP are affected by the environmental conditions of lakes, which in turn alter the mechanisms of the P supply to the water column [9,10]. Especially in eutrophic lakes, phytoplankton may play an important role in PP cycling in aquatic environments. The distribution of PP changes accordingly during the process of phytoplankton growth or the dead phytoplankton debris returning to the water column due to sediment re-suspension. Furthermore, phytoplankton blooms can alter the pH and dissolved oxygen (DO) of lake water [11]. Therefore, the effect of lake environmental factor on the biogeochemical cycling of PP and the contribution of different phosphorus species to eutrophication in aquatic systems deserve detailed study.

The content and species of P in lake water and their relationship with water eutrophication have attracted great attention. Most of the previous studies have focused on P in surface water and sediments [11,12], while P in the SPM has received much less attention [13]. This limited knowledge restricts our understanding of the role of PP in lake eutrophication. Thus, it is necessary to characterize the temporal and spatial variations of PP species and their contributions to eutrophication. In this paper, our major objectives were: (1) to quantify the spatial and temporal variations of PP species in different ecotypes of Lake Taihu, a large shallow, eutrophic lake in China, (2) to elucidate the relationships between the species of PP and associated water environmental factors, and (3) to evaluate the contributions to eutrophication from different PP species.

## 2. Materials and Method

### 2.1. Study Area and Sample Collection

Lake Taihu, located in the Yangtze River Delta (30°55′42″–31°33′50″ N, 119°53′45″–126°36′15″ E) is the second largest freshwater lake in China. It covers an area of 2338 km^2^ and is a typical shallow lake with an average water depth of approximately 2 m. It is an important resource for drinking water, shipping, freshwater aquaculture, and farming [14]. As a result of more than one hundred inflow and outflow rivers around Lake Taihu, which receive a large amount of phosphorus-containing wastewater from point source (industrial effluent and municipal sewage) and non-point source (mainly agricultural wastewater), the lake has been heavily contaminated [15]. About 70.7% of the annual input of total phosphorus was derived from the northwest zone of the catchment [16], which resulted in a eutrophic and partially hyper-eutrophic state of the lake water. Even though the water quality of the lake has improved partially in recent years after a long period of eutrophication control, sporadic and seasonal cyanobacterial blooms in Lake Taihu remain frequent.

**Figure 1:**
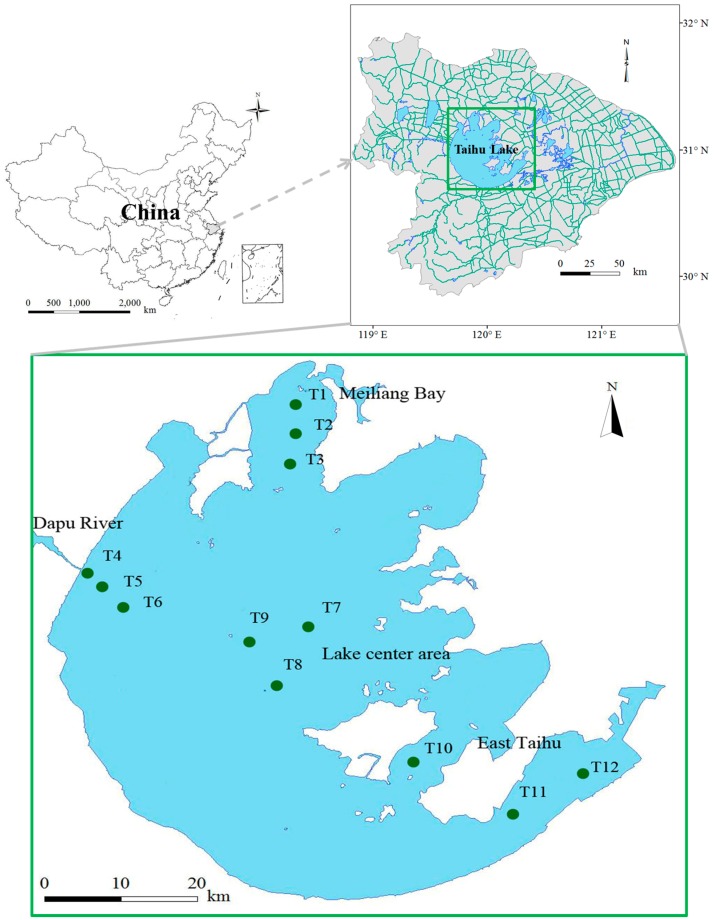
Sampling sites in Lake Taihu: Sites T1, T2 and T3 in Meiliang Bay represent a phytoplankton-dominated zone; Sites T4, T5 and T6 in Dapu River estuary represent an estuary zone; Sites T7, T8 and T9 in the central area of Lake Taihu represent the lake center zone; and Sites T10, T11 and T12 represent a macrophyte-dominated zone.

Twelve representative sites were selected in triplicate in four zones of Lake Taihu with different ecotypes. These were classified into a phytoplankton-dominated zone (sites T1, T2 and T3), estuary zone (sites T4, T5 and T6), lake center zone (sites T7, T8 and T9), and macrophyte-dominated zone (sites T10, T11 and T12) (Figure 1). The water samples were collected seasonally during 2014–2015 (summer: August 2014; autumn: November 2014; winter: February 2015; spring: May 2015), which covered periods of rapid propagation, growth and decomposition of phytoplankton. Samples were collected once each season.

Surface water (~0.5 m depth) was collected directly into acid-cleaned HDPE plastic bottles using stainless steel hydrophore. Depending on the suspended particle concentration, between 15–20 L of water samples were stored in a cooler with ice and transferred to the laboratory for further analysis.

### 2.2. Sample Pretreatment and Preparation

Samples of approximately 10 L of lake water were filtered using pre-combusted 0.7 μm GF/F filters (Whatman) for PP analysis. The filtrates were mainly used for dissolved P analysis. Samples of approximately 1 L of lake water were collected and filtered using pre-combusted and weighed 0.7 μm GF/F filters (Whatman, Meterstone, Britain) for suspended particulate matter (SPM) and particulate organic matter (POM) analysis. The subsamples without filtration were measured for total P (TP).

### 2.3. Physical and Chemical Index Analysis

Water transparency was measured by means of Secchi disc. Dissolved oxygen (DO) was measured by a dissolved oxygen meter (LDOTM, HACH, Loveland, CO, USA). pH was measured using a pH meter (HQ11d, HACH, Loveland, CO, USA). Suspended particulate matter (SPM) was obtained through drying (105 °C for 4 h) and weighing. POM was calculated from the loss on ignition (450 °C for 4 h) [17]. TP concentration in water was analyzed at 700 nm by the colorimetric technique after 30 min of autoclave mediated digestion (120 °C with K_2_S_2_O_8_ and NaOH) of unfiltered and filtered samples, respectively [18]. Soluble reactive phosphorus (SRP) concentration in the water was measured using the molybdenum blue method [19]. For PP concentrations in the SPM, the Nuclepore filter samples were wetted with 0.5 M MgCl_2_ solution and heated in an oven at 95 °C until dry, followed by ashing in a furnace at 550 °C for 2 h to decompose organic P compounds [20]. The residue was extracted using 1 M HCl solution at room temperature for 24 h. PIP was determined by direct extraction from filter samples in 1 M HCl solution at room temperature for 24 h [21]. After neutralization and dilution, both PP and PIP extractions were analyzed, and the concentration of POP was then calculated from the difference between PP and PIP. Chlorophyll *a* was measured spectrophotometrically at 750 and 665 nm after extraction of phytoplankton biomass concentrated on GF/C filters (Whatman, Meterstone, Britain) using 90% hot ethanol [22]. Three parallel samples were analyzed for the above parameters.

### 2.4. Molecular PP Analysis by ^31^P NMR

PP samples were extracted in 50 mL acid-washed falcon tubes with 15 mL each of 0.5 M NaOH and 0.1 M EDTA for 16 h at room temperature on a shaking plate, followed by centrifugation at 2000 rmp for 20 min [23]. NaOH and EDTA extraction for PP were concentrated to approximately 0.5 mL in a rotary vacuum evaporator at 28 °C for solution ^31^P NMR spectroscopy analysis [13].

Before the ^31^P NMR analysis, the extracts were transferred into a 5 mm NMR tube and D_2_O was added into the supernatant to reach 10% proportion for signal lock [13]. The ^31^P NMR spectra were measured at 161.84 MHz on a Bruker AV400 spectrometer (Bruker, Billerica, MA, USA) equipped with a 5-mm broadband probe, using a 90 pulse, relaxation delay 2 s and acquisition time 0.5 s, acquiring around 19,000 transients.

The solution ^31^P NMR spectra were obtained at 161.84 MHz on an AV 400 spectrometer equipped with a 5-mm broadband probe, using a 90° pulse, relaxation delay 2 s and acquisition time 0.5 s, acquiring around 19,000 transients. Chemical shifts were recorded relative to 85% H_3_PO_4_ via the signal lock [24]. Peak assignments were made using a ^31^P NMR chemical shift of Ortho-P (6–7 ppm), Mono-P (4–6 ppm), Diester-P (0–3 ppm), Pyro-P (−3.5 to −4.5 ppm), Poly-P (−17 to −19 ppm) and phosphonate (18–20 ppm) [25]. The peak area was calculated by integration, and the spectra were plotted with a line broadening of 3 Hz.

### 2.5. Statistical Analysis

The experimental data were analyzed using SPSS 19.0 for Windows (IBM, Armonk, NY, USA) and Origin 8.5 (OriginLab, Northampton, MA, USA). Pearson’s correlation coefficient was calculated to reveal the relationship between *P* speciation and various water quality parameters. All analysis was performed using standard procedures in Microsoft Excel (Microsoft, Redmond, WA, USA).

## 3. Results

### 3.1. General Characteristics of Overlying Water

The general characteristics of overlying water in four lake zones of Lake Taihu with different ecotypes are shown in Table 1. Water transparency ranged from 0.2 m to 1.7 m, the highest value of transparency appeared in February in the macrophyte-dominated zone and the lowest value of transparency appeared in the estuary zone. The average concentration of DO ranged from 6.90 to 9.84 mg/L, with the highest level appearing in macrophyte-dominated zone and the lowest in estuary zone. The variation trend in the pH value was similar to DO, with the highest values appearing in the macrophyte-dominated zone (8.58) and the lowest in the estuary zone (8.04).

The average concentration of SPM in the estuary zone was 55.25 mg/L, and the concentration of SPM in the phytoplankton-dominated zone was much lower (36.73 mg/L) than in other three zones. The distribution characteristics of POM concentration were as follows: phytoplankton-dominated zone (17.46 mg/L) > lake center zone (12.13 mg/L) > estuary zone (10.13 mg/L) > macrophyte-dominated zone (7.15 mg/L). The ratio of POM to SPM ranged from 13.68 to 47.30 mg/L, with the highest ratio appearing in the phytoplankton-dominated zone and the lowest in the macrophyte-dominated zone. The concentration of Chl *a* ranged from 9.84 to 52.13 μg/L, and its concentration trend was the same as that of SPM. The average concentration of TP ranged from 0.05 to 0.22 mg/L, with the highest levels appearing in the estuary zone and the lowest in the macrophyte-dominated zone. Similar to TP concentration, the highest concentration of SRP occurred in the estuary zone (0.076 mg/L), and the lowest concentration occurred in the macrophyte-dominated zone (0.004 mg/L).

### 3.2. The Composition and Variation Characteristics of PP

#### 3.2.1. The Spatial and Temporal Distribution of PP, POP and PIP

**Figure 2:**
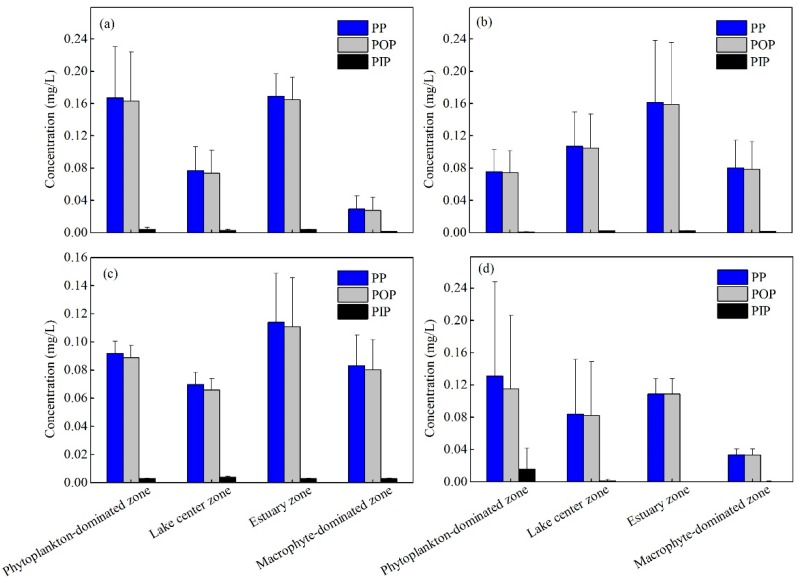
The temporal and spatial variability of particulate P (PP), particulate inorganic P (PIP) and particulate organic P (POP): (**a**) autumn (November 2015); (**b**) winter (February 2015); (**c**) spring (May 2016); (**d**) summer (August 2016).

The spatial and temporal distribution of PP concentration in the four zones of Lake Taihu are shown in Table S1 and Figure 2. The average concentration of PP ranged from 0.034 to 0.169 mg/L, with the highest levels appearing in the estuary zone and the lowest in the macrophyte-dominated zone. Similar to PP concentration, the highest concentration of POP occurred in the estuary zone (0.165 mg/L), and the lowest concentration occurred in the macrophyte-dominated zone (0.033 mg/L). Different from the spatial distribution of PP and POP, the highest value of PIP occurred in the phytoplankton-dominated zone (0.016 mg/L). In the phytoplankton-dominated zone, the concentration of PP and POP in summer was significantly higher (*p* < 0.05) than in other seasons, while the highest concentration of PIP occurred in spring (0.016 mg/L), which is much higher than that in other seasons. In the lake center zone, the concentration of PP, POP and PIP showed no significant seasonal difference (0.077–0.107 mg/L, 0.074–0.105 mg/L, and 0.001–0.004 mg/L, respectively), the concentration of PP and POP in autumn is slightly higher than that in the other seasons, while the highest concentration of PIP occurred in winter. In estuary zone, the highest concentration of PP, POP and PIP all occurred in summer (0.169 mg/L, 0.165 mg/L and 0.004 mg/L, respectively). In the macrophyte-dominated zone, the concentration of PP and POP in autumn and winter were higher than that in spring and summer. The concentration of PIP showed no significant seasonal difference, and its concentration in winter is slightly higher compared to the other seasons.

#### 3.2.2. Molecular Species of PP Measured by ^31^P NMR

**Figure 3:**
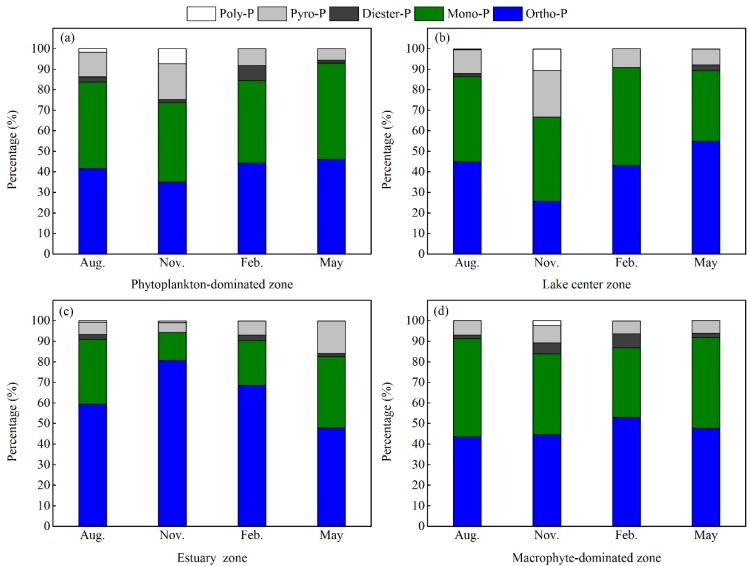
Percentages of each PP species in Lake Taihu: (**a**) autumn (November 2015); (**b**) winter (February 2015); (**c**) spring (May 2016); (**d**) summer (August 2016).

Extracted particulate P in NaOH-EDTA ranged from 0.020 mg/L to 0.154 mg/L, with an extraction efficiency of 37–99% (Table S2). The species of particulate P mainly consisted of Ortho-P, Mono-P, Diester-P, Pyro-P and Poly-P. (Figure 3).

Ortho-P and Mono-P were the dominant species in the four lake zones (Ortho-P: 25.7–80.6%; Mono-P: 13.7–47.8%), followed by Pyro-P (4.8–22.6%), and the sum of the proportions of Ortho-P, Mono-P and Pyro-P in TPP was higher than 72.6% (Figure 3 and Table S2). Ortho-P in the estuary zone is significantly higher than that in other lake zones while the higher concentration of Mono-P and Pyro-P occurred in the phytoplankton-dominated zone and lake center zone.

Temporal variations of the proportions of the main species of Ortho-P, Mono-P and Pyro-P were appreciable, but they were not the same in different zones. The maximum of Ortho-P was present in May in the four lake zones, except for the estuary zone, (phytoplankton-dominated zone: 46.1%; lake center zone: 54.9%; macrophyte-dominated zone: 47.6%) while the maximum of Ortho-P was in November in estuary zone (80.6%). The maximum of Mono-P was in May in the phytoplankton-dominated zone (47.0%) and the estuary zone (34.9%). The maximum of Mono-P was in August in the macrophyte-dominated zone (47.0%). The maximum of Mono-P was in February in the lake center zone (47.8%). The maximum of Pyro-P was present in November in four zones, except for the estuary zone (phytoplankton-dominated zone: 17.5%; lake center zone: 22.6%; macrophyte-dominated zone: 8.5%) while the maximum of Pyro-P was in May in the estuary zone (15.8%). The concentration of Poly-P, which only occurred in August and November, was relatively high in the phytoplankton-dominated zone (peak value of 7.3%) and lake center zone (peak value of 10.5%). The concentrations of Pyro-P+Poly-P accounted for 5.3–33.1% of NaOH-EDTA TP extracted from PP.

### 3.3. Temporal Variations in the Relationships among PP Species Concentration and Associated Water Environmental Factors

**Figure 4:**
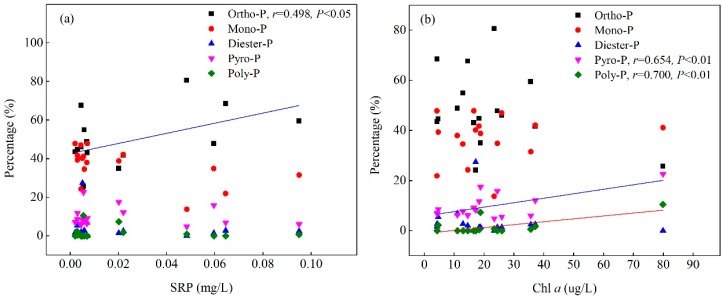
Correlation analysis of NMR P concentrations in PP with (**a**) soluble reactive phosphorus (SRP) and (**b**) chlorophyll *a* (Chl *a*) in Lake Taihu.

The correlation between PP species and water environment factors (Chl *a* and SRP) are shown in Figure 4. Among all the PP species, only Ortho-P was positively correlated with SRP concentrations (*r* = 0.498, *p* < 0.05), no significant relationship was observed between the other PP species and SRP concentrations. Different from the above, Pyro-P and Poly-P were significantly positively correlated with Chl *a* (*p* < 0.01), while no correlation relationship was found between the other PP species and Chl *a*.

## 4. Discussion

### 4.1. Spatial and Temporal Distribution of PP and Its Environmental Influencing Factors

The concentrations of PP were significantly higher in the water of the phytoplankton-dominated zone and estuary zone where cyanobacteria bloom frequently occurs, especially during the summer (Figure 2 and Table S1). For the estuary zone in summer, the SPM carried by inflowing rivers is the main source of particulate phosphorus, and the difference in concentration of SPM and the river flow and runoff flux are the main reason for the seasonal variation of PP. While for the phytoplankton-dominated zone in summer, the growth and reproduction of phytoplankton was an important factor driving the seasonal variations of PP. These findings could be proved by the positive correlation between PP and POM (*p* < 0.05) and significant correlation between PP and Chl *a* (*p* < 0.01).

POP was the dominant form of PP (it accounted for 88.0–99.9% of PP), and correlation analysis revealed that POP was significantly correlated with TP in the overlying water (*p* < 0.01) (Table 2), which indicates that POP represents a particularly important source of P in Lake Taihu. As shown in Figure 2 and Table S1, the spatial and temporal distribution of POP is consistent with that of PP, significant correlation was found between POP and POM (*p* < 0.01), and POP concentrations were also positively correlated with pH (*p* < 0.05). These results support our hypothesis that changes in the PP concentration in Lake Taihu are controlled by biogenic P rather than by inorganic P supplied by SPM. Furthermore, significant positive correlation was found between PIP and Chl *a* (*p* < 0.01 while there was no significant correlation between POP and Chl *a*, indicating that PIP is more easily used by phytoplankton than POP. Although POP is relatively stable in a water environment and cannot be directly absorbed by phytoplankton, POP is easier to mineralize and decompose under alkaline water and high temperature conditions [26,27]. This means that the mineralization of POP could be an important source of P in phytoplankton-dominated zone in summer.

### 4.2. Spatial and Temporal Distribution of Molecular Species of PP

Results from ^31^P NMR analyses showed that Ortho-P was the dominant fraction (on average 49.15%) of all PP compound groups detected, which was in accordance with a previous report [28]. The peak of Ortho-P concentrations was found in May in the phytoplankton-dominated zone and lake center zone; this phenomenon demonstrated that the concentration of Ortho-P in these two zones was affected by phytoplankton-derived particulate matter. In the macrophyte-dominated zone, the peak of Ortho-P concentrations occurred in February, suggesting that plant debris had a major contribution to the concentration of Ortho-P. In the estuary zone, the average concentration of Ortho-P was much higher than the other zones, which was mainly affected by input of exogenous particulate matter.

Among the detected organic P compound classes in suspended particles, Mono-P accounted for the largest proportion; this was because Mono-P generally synthesizes as nucleic acids and membrane phospholipids for microbial growth through both de novo and salvage pathways [29,30]. Many studies have identified that Mono-P mainly includes inositol hexakisphosphate, mononucleotides and sugar phosphates, and inositol hexakisphosphate is relatively stable in environmental samples [31,32].

Based on previous studies [33,34,35], the inorganic P, including Pyro-P and Poly-P is more labile than other P compounds, because of their shorter half-lives, which readily increases bioavailable P in the water column. The concentration of (PyroP+PolyP) in PP was relatively high in the phytoplankton-dominated zone and lake center zone in August and November, which indicated that the source of particulate (Pyro-P+Poly-P) could be living algae or algae residues enriched in Pyro-P [36]. The content of Poly-P was below the limit of detection in February and May, which may result from rapid biological transformation.

### 4.3. Potential Contribution of Various PP Species to Lake Eutrophication

The content and bioavailability of different species of PP vary greatly in in lake ecosystems, which determines their level of contribution to eutrophication [37,38]. Correlation analysis showed that there was a significant positive correlation between Ortho-P and SRP (*p* < 0.01) (Figure 4a), and previous research has showed that Ortho-P is easy to release from SPM and be used by algae growth [39]. As shown in Figure 4b, Pyro-P and Poly-P were positively correlated with Chl *a* (*p* < 0.01). Previous studies found that Pyro-P and Poly-P in SPM were significantly correlated with phytoplankton biomass [23], and Poly-P and Pyro-P can be accumulated intracellularly by all organisms as energy or nutrient storage molecules [40]. That means when the content of dissolved phosphorus is insufficient in the water, Ortho-P, Pyro-P and Poly-P are be rapidly degraded and provide available P sources for further breeding of phytoplankton during the period of algal blooms. Combined with the higher content of Ortho-P, Pyro-P and Poly-P in hyper-eutrophic zones, the decomposition and release of particulate phosphorus can maintain long-term eutrophication even if the dissolved phosphorus in water is at a low level.

## 5. Conclusions

In summary, the spatial and temporal distribution of different PP species varied appreciably in Lake Taihu with different ecotypes. POP was the dominant form of PP (>88.0%). The concentrations of POP showed higher levels in hyper-eutrophic zones during warm seasons, and phytoplankton blooms and input of exogenous particulate matter were the main sources of POP in lake water. Ortho-P was the dominant molecular species of PP and positively correlated with SRP (*p* < 0.01), which suggested that the release of Ortho-P from SPM was the main source of SRP in the lake water. Pyro-P, which is regarded as a highly labile species of P compounds, represented a large fraction of the PP, and its significant positive correlations with Chl *a*, indicating that the concentration of Pyro-P could be used as an important indicator for the degree of eutrophication of Lake Taihu. These results proved that PP in lake water is a significant factor supporting lake eutrophication and must be controlled, especially in the bloom sensitive northwestern zone (phytoplankton-dominated zone and estuary zone). Further investigation is needed to understand the PP degradation and ecological effects of phytoplankton-derived PP in the lake environment.

## Figures and Tables

**Table 1:** The variation of physical and chemical parameters in different ecological type zones of Lake Taihu: transparency (SD), dissolved oxygen (DO), suspended particulate matter (SPM), particulate organic matter (POM), total phosphorus (TP), soluble reactive phosphorus (SRP), chlorophyll *a* (Chl *a*).

Sample Sites	SD (m)	DO (mg/L)	pH	SPM (mg/L)	POM/SPM (%)	POM (mg/L)	TP (mg/L)	SRP (mg/L)	Chl *a* (μg/L)
Phytoplankton-dominated zone	0.41 ± 0.16	9.84 ± 2.33	8.58 ± 0.18	36.73 ± 30.68	47.30 ± 57.15	17.46 ± 17.48	0.11 ± 0.05	0.014 ± 0.010	52.13 ± 80.45
Estuary zone	0.35 ± 0.12	9.33 ± 2.26	8.35 ± 0.25	55.25 ± 34.22	18.34 ± 10.59	10.13 ± 3.62	0.22 ± 0.05	0.076 ± 0.02	19.25 ± 10.91
Lake center zone	0.24 ± 0.08	6.90 ± 3.58	8.04 ± 0.31	53.97 ± 30.26	22.48 ± 15.18	12.13 ± 4.59	0.13 ± 0.08	0.019 ± 0.024	39.53 ± 44.56
Macrophyte-dominated zone	0.69 ± 0.42	9.60 ± 2.32	8.25 ± 0.37	52.24 ± 79.70	13.68 ± 8.29	7.15 ± 6.61	0.05 ± 0.03	0.004 ± 0.002	9.84 ± 4.90

**Table 2:** Correlation between different particulate phosphorus and environmental factors.

Parameters	SD	DO	pH	SPM	POM	TP	Chl *a*	PP	POP	PIP
**SD**	1.000	0.315 *	0.344 **	0.173	−0.046	−0.350 **	−0.217	−0.176	−0.175	−0.102
**DO**		1.000	0.701 **	0.237	0.276 *	−0.155	−0.035	0.056	0.034	0.219
**pH**			1.000	0.175	0.264 *	0.121	−0.029	0.294 *	0.295 *	0.134
**SPM**				1.000	0.522 **	0.281 *	0.179	0.290 *	0.277 *	0.260
**POM**					1.000	0.298 *	0.731 **	0.614 **	0.550 **	0.853 **
**TP**						1.000	0.377 **	0.689 **	0.707 **	0.186
**Chl *a***							1.000	0.50 8 **	0.243	0.816 **
**PP**								1.000	0.995 **	0.530 **
**POP**									1.000	0.445 **
**PIP**										1.000

* Correlation is significant at *p* = 0.05. ** Correlation is significant at *p* = 0.01.

## Supplementary Materials

The following are available online at http://www.mdpi.com/1660-4601/15/11/2355/s1, Table S1: Spatial and temporal variation of concentrations of particulate P in Lake Taihu, Table S2: Spatial and temporal variation of percentage of PP species in Lake Taihu.
