# Supplementary material for: Spatial and Temporal Distribution of Particulate Phosphorus and Their Correlation with Environmental Factors in a Shallow Eutrophic Chinese Lake (Lake Taihu)
Source: Int J Environ Res Public Health. 2018 Oct 25;15(11):2355. doi: 10.3390/ijerph15112355 (PMC6266604; doi:10.3390/ijerph15112355)
Supplement: Supplementary file 1 [file ijerph-15-02355-s001.docx]

Supplementary

Spatial and Temporal Distribution of Particulate Phosphorus and Their Correlation with Environmental Factors in a Shallow Eutrophic Chinese Lake (Lake Taihu)

Ming Kong, Jianying Chao, Wei Zhuang, Peifang Wang, Chao Wang, Jun Hou, Zhaoshi Wu, Longmian Wang, Guang Gao and Yu Wang

**Table S1.** Spatial and temporal variation of concentrations of particulate P in Lake Taihu.

| **Study Area** | **Season** | **PP mg/L** | **POP mg/L** | **PIP mg/L** | **POP/PP** | **PIP/PP** |
| --- | --- | --- | --- | --- | --- | --- |
| Phytoplankton-dominated zone | August | 0.167 | 0.163 | 0.004 | 0.976 | 0.024 |
|  | November | 0.076 | 0.074 | 0.001 | 0.986 | 0.014 |
|  | February | 0.092 | 0.089 | 0.003 | 0.967 | 0.033 |
|  | May | 0.131 | 0.115 | 0.016 | 0.880 | 0.120 |
| Estuary zone | August | 0.169 | 0.165 | 0.004 | 0.976 | 0.024 |
|  | November | 0.161 | 0.159 | 0.002 | 0.986 | 0.014 |
|  | February | 0.114 | 0.111 | 0.003 | 0.974 | 0.026 |
|  | May | 0.109 | 0.109 | 0.000 | 0.999 | 0.001 |
| Lake center zone | August | 0.077 | 0.074 | 0.003 | 0.962 | 0.038 |
|  | November | 0.107 | 0.105 | 0.002 | 0.980 | 0.020 |
|  | February | 0.070 | 0.066 | 0.004 | 0.945 | 0.055 |
|  | May | 0.084 | 0.083 | 0.001 | 0.983 | 0.017 |
| Macrophyte-dominated zone | August | 0.131 | 0.115 | 0.001 | 0.880 | 0.120 |
|  | November | 0.084 | 0.083 | 0.001 | 0.983 | 0.017 |
|  | February | 0.109 | 0.109 | 0.000 | 0.999 | 0.001 |
|  | May | 0.034 | 0.033 | 0.000 | 0.987 | 0.013 |

**Table S2.** Spatial and temporal variation of percentage of PP species in Lake Taihu.

| **Study Area** | **Season** | **Ortho-P %** | **Mono-P %** | **Diester-P %** | **Pyro-P %** | **Poly-P%** | **NMR TP mg/L** | **Extraction Efficiency %** | **Pi %** | **Po %** |
| --- | --- | --- | --- | --- | --- | --- | --- | --- | --- | --- |
| Phytoplankton-dominated zone | August | 41.7 | 42.1 | 2.5 | 12.1 | 1.7 | 0.109 | 58 | 55.4 | 44.6 |
|  | November | 35 | 38.8 | 1.4 | 17.5 | 7.3 | 0.045 | 73 | 59.8 | 40.2 |
|  | February | 44.2 | 40.2 | 7.4 | 8.2 | — | 0.070 | 69 | 52.4 | 47.6 |
|  | May | 46.1 | 47.0 | 1.4 | 5.5 | — | 0.033 | 51 | 51.6 | 48.4 |
| Lake center zone | August | 44.8 | 41.7 | 1.3 | 11.7 | 0.4 | 0.071 | 81 | 57.0 | 43.0 |
|  | November | 25.7 | 41.1 | — | 22.6 | 10.5 | 0.102 | 66 | 58.9 | 41.1 |
|  | February | 43.1 | 47.8 | — | 9.1 | — | 0.043 | 71 | 52.2 | 47.8 |
|  | May | 54.9 | 34.6 | 2.7 | 7.7 | — | 0.032 | 65 | 62.6 | 37.4 |
| Estuary zone | August | 59.5 | 31.5 | 2.4 | 6.0 | 0.6 | 0.086 | 49 | 66.1 | 33.9 |
|  | November | 80.6 | 13.7 | 0.0 | 4.8 | 0.8 | 0.126 | 71 | 86.3 | 13.7 |
|  | February | 68.5 | 21.9 | 2.7 | 6.8 | — | 0.046 | 59 | 75.3 | 24.7 |
|  | May | 47.8 | 34.9 | 1.4 | 15.8 | — | 0.033 | 37 | 63.6 | 36.4 |
| Macrophyte- dominated zone | August | 43.5 | 47.8 | 1.7 | 7.0 | — | 0.014 | 76 | 50.4 | 49.6 |
|  | November | 44.6 | 39.3 | 5.4 | 8.5 | 2.2 | 0.099 | 84 | 55.4 | 44.6 |
|  | February | 52.8 | 34.0 | 6.8 | 6.3 | — | 0.060 | 99 | 59.1 | 40.9 |
|  | May | 47.6 | 44.3 | 2.0 | 6.1 | — | 0.020 | 79 | 53.6 | 46.4 |

© 2018 by the authors. Submitted for possible open access publication under the terms and conditions of the Creative Commons Attribution (CC BY) license (http://creativecommons.org/licenses/by/4.0/).
